# The potential of VKORC1 polymorphisms in *Mustelidae* for evolving anticoagulant resistance through selection along the food chain

**DOI:** 10.1371/journal.pone.0221706

**Published:** 2019-08-29

**Authors:** Matthias Stöck, Florian Reisch, Morten Elmeros, Doreen Gabriel, Werner Kloas, Eva Kreuz, Pia Lassen, Alexandra Esther

**Affiliations:** 1 Department of Ecophysiology and Aquaculture, Leibniz-Institute of Freshwater Ecology and Inland Fisheries, Berlin, Germany; 2 Institute of Biochemistry, Universitätsmedizin Berlin Charité, Berlin, Germany; 3 Department of Bioscience, Aarhus University, Rønde, Denmark; 4 Institute for Crop and Soil Science, Julius Kühn Institute, Federal Research Centre for Cultivated Plants, Braunschweig, Germany; 5 Department of Environmental Science, Aarhus University, Aarhus, Denmark; 6 Institute for Plant Protection in Horticulture and Forests, Julius Kühn Institute, Federal Research Centre for Cultivated Plants, Münster, Germany; University of Iceland, ICELAND

## Abstract

In response to strong selection, new mutations can arise quickly and sweep through populations, particularly, if survival and reproduction depend on certain allele copies for adaptation to rapidly changing environments, like resistance against deadly diseases or strong toxins. Since the 1950s, resistance to anticoagulant rodenticides in several rodents has emerged through single nucleotide mutations in the vitamin-K-epoxid-reductase-complex-subunit-1 (VKORC1) gene, often located in its exon 3. Detection of high prevalence and concentrations of anticoagulant rodenticides in non-target vertebrates, including carnivorous Mustelidae, let us assume that secondary exposure by feeding on poisoned prey may also cause selection along the food chain and we hypothesized that VKORC1-based resistance might also have evolved in rodents’ predators. Using newly-developed mustelid-specific primers for direct sequencing of genomic DNA, we studied VKORC1-DNA-polymorphisms in 115 mustelids of five species (*Martes martes*, *M*. *foina*, *Mustela nivalis*, *M*. *erminea*, *M*. *putorius*), obtained from northern Denmark, yielding six sites with nonsynonymous and several synonymous amino acid polymorphisms in exon 3. Comparison of these VKORC1-genotypes with hepatic rodenticide residues (obtained by HPLC combined with fluorescence or mass spectrometry) in 83 individuals (except *M*. *martes*), using generalized linear models, suggested that anticoagulant levels depended on species and specific polymorphisms. Although most VKORC-1 polymorphisms may present standing genetic variation, some are situated in resistance-mediating membrane parts of the VKORC1-encoded protein, and might be a result of selection due to exposure to anticoagulant poisons. Our new molecular markers might allow detecting indirect effects of anticoagulant rodenticides on rodent predator populations in the future.

## Introduction

Under common selection scenarios of complex environments, evolutionary changes may gradually accumulate by subtle polygenic adaptation [e.g. 1,2]. However, in response to strong selection, new mutations can arise quickly and sweep through populations [1,2,3]. This is especially expected if survival and reproduction depend on the possession of certain allele copies, like for adaptation to rapidly changing environments [4], such as resistance against deadly diseases [5] or strong toxins [6], as for example, the application of strong rodenticides. While gene variants arising after such deadly impacts may rarely stem from *de novo* mutations, the majority of these polymorphisms is thought to rather arise from standing genetic variation due to allele frequency changes and may be accompanied by hard or soft selective sweeps [1,4,7].

Shortly after isolation of 4-hydroxycoumarins from spoiled sweet clover hay (*Melilotus officinalis*), identification and synthesizing (e.g. dicoumarol [8,9]), coumarin derivatives such as warfarin have been developed to lower blood coagulation and clogging for both therapeutic applications [10,11] and rodent control [12,13]. Metabolized coumarin derivatives affect blood coagulation in vertebrates by inhibition of the vitamin K epoxide reductase complex subunit 1 (VKORC1) [14,15,16], a small transmembrane protein of the endoplasmic reticulum of most eukaryotes [17,18]. It recycles vitamin K 2,3-epoxide to vitamin K hydroquinone, which is essential for the posttranslational γ-carboxylation of many blood coagulation factors [17]. In humans, VKORC1 gene consists of three exons with 6126 base pairs and codes for the VKORC1 protein, comprising 163 amino acids.

Since the late 1950s, resistance against warfarin-containing (later on so-called 1^st^ generation ARs) rodenticides has emerged in several *Rattus norvegicus* and *Mus musculus* populations in Europe [19, 20, 21] but remained mechanistically unexplained. In 2004, when examining human disorders (e.g., VKCFD2), mutations in the membrane-imbedded part of the VKORC1 protein, were demonstrated to induce coumarin resistance by these haplotypes [14,18, 22]. Since about that time, missense mutations in exon 3 of VKORC1, have been shown to encode warfarin resistance in several *Rattus* species [23,24,25,26], in *Mus musculus* [27,28] as well as in *Mastomys* mice [29] (S1 Table). In rodents, the best-characterized mutations are the amino acid (AA) replacements Tyr139Cys, Tyr139Phe and Leu120Glu, known to mediate resistance to all first-generation and to some of the second-generation anticoagulant rodenticides (i.e. bromadiolone and difenacoum) [30].

The adaptive alleles in *R*. *norvegicus* have been shown to segregate in the ancestral environment [31] and formed the genetic basis for rapid resistance evolution against warfarin with several natural allelic VKORC1 variants conferring resistance. These alleles occurred in different populations of brown rats throughout Europe as part of their standing genetic variation [7,26,31,32]. Wild resistant rodents have been shown to bear higher residues in their livers, supposedly rather based on the prolonged survival time than on accumulation of anticoagulant rodenticides [33–34].

Resistance caused the application of the more toxic and persistent second-generation rodenticides (i.e. brodifacoum, flocoumafen and difethialon), which involve higher exposure risks for non-target mammals and birds [35,36,37]. Since decades, high anticoagulant rodenticide (AR) residues (used here and throughout the paper in the sense of “traces” or “concentrations”) have been detected in predators of rodents, particularly carnivores of the *Mustelidae* family (e.g., UK [38,39]; France [40]; USA [41]). In our focal research area, Denmark, rats, a potential prey of mustelids, are declared a notifiable species and systematically regulated by professional pest controllers, involving intense use of anticoagulants [42]. Furthermore, until 2012 anticoagulants were used to control voles in forests, and until 2014, private people could use AR s to kill mice in Denmark. Anticoagulant rodenticide resistance, facilitated by Tyr139Cys, has been frequently detected in *R*. *norvegicus* [42,43]. In rodents, resistance has not been reported from the anticoagulants brodifacoum and flocoumafen, used in Denmark, but are known for coumatetralyl, bromadiolone and difenacoum [27].

Residues have been detected in 97% of 61 stoats (*Mustela erminea*), 95% of 69 weasels (*Mustela nivalis*), 94% of 69 polecats (*Mustela putorius*) and 98% in 71 stone martens (*Martes foina*) [44,45]. In all four species, animals with hepatic concentrations of bromadiolone higher than 1000 ng/g ww (wet weight) were recorded, particularly in *Martes foina*. A subset of these animals was also used in our study (see Materials and Methods). Mustelids are highly susceptible to ARs [45,46, 47]. High concentrations might be due to recent ingestion of a high dosage of the rodenticides. However, since regulations command baits to be placed inaccessibly for non-target species, rodenticide residues in mustelids most likely originate from feeding on poisoned prey, even in food generalists as the genus *Martes* [44,45]. Therefore, the question arose whether resistance, similar to that evolved in their rodent prey may also be acquired through poison transfer along the food chain in the mustelid predators.

Thus, in the present study, we examine the prevalence of VKORC-1 non-synonymous substitutions in mustelids that are exposed to rodenticides. As previously shown (see Discussion), these mustelid species largely experience secondary pesticide exposure, most probably via predator-prey-interactions in food webs. To understand this, we have i) developed molecular markers with focus on exon 3 of VKORC1 and ii) identified polymorphisms in five free-living mustelid species. Based on great evolutionary conservation of VKORC1 among mammals, we assumed variation to occur potentially also as recessive heterozygotes and thus standing genetic variation that may counterbalance potentially negative mutational effects as documented in rodents. The co-measured residues from individual mustelids allowed us iii) examining whether VKORC1 polymorphisms may show relationships to AR-loads. We aimed at evaluating the potential of VKORC1 polymorphisms as indicators for ongoing or future evolution of resistance to coumarine-derivative ARs in mustelids.

## Materials and methods

### Samples

**Fig 1:**
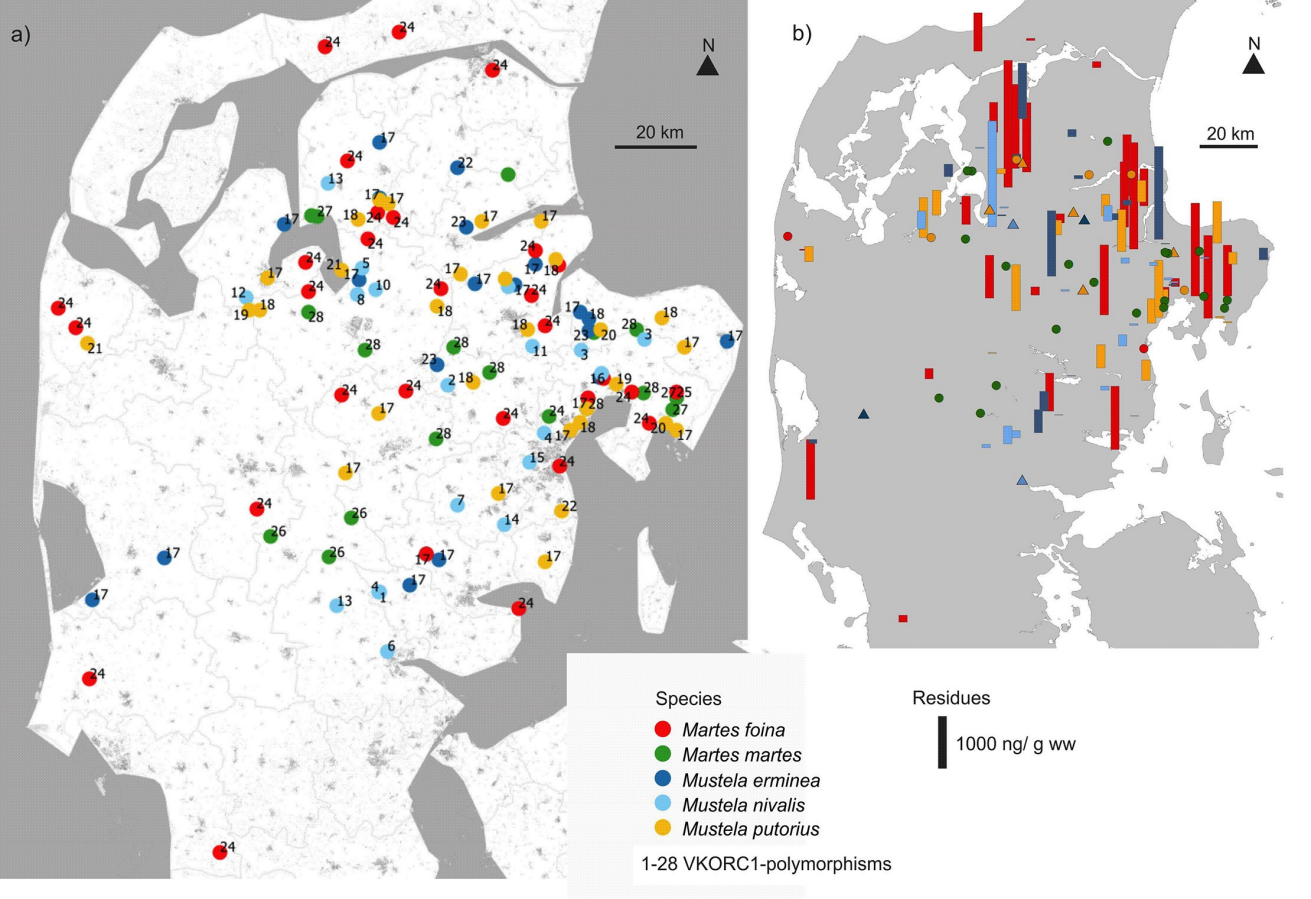
**a)** Sampling sites and VKORC1-polymorphisms for the five mustelid carnivores in Denmark (map space approximately 55°–57° N, 8°–10° E). **b)** Individual hepatic AR residue amounts (sum of the residues of coumatetralyl, bromadiolone and difenacoum), indicated by bar heights; triangles indicate sampling sites for mustelids with no detectable AR residues (0 ng/g wet weight) in their liver; dots in (panel b) indicate sampling sites for individuals, which were not included in the AR-measurements [44,45].

We have studied five mustelid species based on initially 120 samples, comprising 20 *Martes martes*, 30 *Martes foina*, 20 *Mustela nivalis*, 20 *Mustela erminea* and 30 *Mustela putorius*. The mustelid carcasses were collected between 2000 and 2013 in Denmark (55°–57° N, 8°–10° E; Fig 1). Date, cause of death (mostly road kills, predation and culling), location and geographic coordinates (S2 Table) were recorded for each individual. Liver or muscle samples of all animals were stored frozen. For the DNA analyses subsamples were stored in 96% ethanol at room temperature. Due to insufficient DNA quality, VKORC1-genotypes could be only obtained from 115 samples (Results).

### Residues analyses

To examine potential relationships with VKORC1 genotypes, we included published hepatic AR-data of the same individual mustelids (S3 Table). In two previous studies [44,45], the concentration of five regionally relevant ARs (i.e. coumatetralyl, bromadiolone, difenacoum, brodifacoum and flocoumafen) was analyzed in 92 individuals from four species (except *Martes martes*). *Martes martes* was not included in these former studies and thus, residue data were unavailable. In *Mustela nivalis* and *M*. *erminea*, residues in liver tissue (sample size mean wet weight ± standard error: 0.59 g ± 0.28 g and 0.99 g ± 0.29 g) were determined by high pressure liquid chromatography (HPLC) coupled with a fluorescence and photodiode array detector [48]. For details on extraction, clean-up and quality assurance procedures, see [44]. For our study, AR-concentrations in *Mustela erminea* and *M*. *nivalis* were corrected for the average recovery rates per substance (coumatetralyl 51%, bromadiolone 81%, difenacoum 75%, brodifacoum 78% and flocoumafen 78%; [44]). Recovery rates were assessed from spiked control samples of chicken liver with known rodenticide concentrations.

In *Mustela putorius* and *Martes foina*, rodenticide residue levels were also determined in liver tissues (sample size mean wet weight ± standard error: 0.52 g ± 0.01 g and 0.53 g ± 0.03 g) by high pressure liquid chromatography (HPLC) coupled to tandem mass spectrometry (MS-MS) [49]. Concentrations were corrected for recovery rates, for each AR substance (coumatetralyl 77%, bromadiolone 36%, difenacoum 59%, brodifacoum 36% and flocoumafen 50%). Recovery rates were assessed from spiked controls of chicken liver with known rodenticide concentrations (details: [45]). The MS-MS method is more sensitive than the older fluorescence-photodiode array detection method [44,45]. To compare and analyse data from both studies, ARs concentrations in *Martes foina*, *Mustela putorius and M*. *erminea* were adjusted to the highest AR-limits as used to measure AR in *Mustela nivalis* (i.e. values in *Martes foina*, *Mustela putorius* and *M*. *erminea* that were below the detection limits in *M*. *nivalis* were set formally to 0 ng/g wet weight; S3 Table).

### Genetic analyses

Subsamples of liver or muscle were taken and DNA extracted using Puregene Core Kit A (Qiagen) at the Julius Kuehn Institute. Subsequent molecular work (i.e. primer development, PCRs and cloning) was performed at the Leibniz-Institute of Freshwater Ecology and Inland Fisheries (IGB). Primers were developed by aligning DNA sequences of VKORC1, available from GenBank, for mice and rats and few available haplotypes of carnivores, namely panda, dog, cat and that of the only complete mustelid genome *Mustela putorius*, available on Ensemble. Two primer pairs were developed *de novo* (Table 1): DogEx3F and DogEx3R are both exon-based and amplify almost the entire exon 3 (ca. 200 bp) of VKORC1. Primers Must1F/Must1R stretch into the flanking intron 2 and amplify about 500 bp.

**Table 1:** Sequences of the newly developed mustelid-specific primers targeting VKORC1-exon 3 (MustF/R) and a flanking intron (DogEx3F/R).

Primer name	Sequence (5' = >3')	PCR product size
Must1F	GRCCCGCTGGGCATCTAT	ca. 500 bp
Must1R	AGGGTCCCTCRCAGACAGA	
DogEx3F	CCGCTGGGCATCTATCCT	ca. 200 bp
DogEx3R	CAGTGCCCCTTGACCTTG	

PCR reactions comprised volumes of 25 μl with the following ingredients: 9.875 μl of ddH_2_O, 2.5 μl of TopTaq buffer including 1.5 mM MgCl_2_ supplemented with 1 μl of MgCl_2_ solution, 2.5 μl of solution Q, 2.5 μl of dNTP (2.5 mM each), 1.25 μl of each of two primers (in most cases MustF1/MustR1), 0.125 μl of TopTaq polymerase (all ingredients: Qiagen) and usually 4 ul of DNA (of 20–60 ng/μl; roughly quantified using Nanodrop). In case of apparent DNA degradation, we increased the DNA volume to up to 6 ul and reduced the water. Amplifications were performed on Mastercycler® ep gradient (Eppendorf) and comprised 5 min 95°C (denaturation), followed by 40 cycles of [1 min 95°C (denaturation), 1 min 58.1°C (annealing), 1 min 72°C (elongation)], and 5 min 72°C (final elongation). PCR products were visualized on 1.5% agarose gels in 1xTAE buffer electrophorese, running at 80 V for 40 min, using ethidium bromide and the GelDoc^TM^ XR+ system, and then directly Sanger-sequenced with the forward primers.

Due to low DNA quality, presumably caused by degradation in road kills, in several cases, bands of weak PCR-products were entirely cut from Low Melting Plaque Agarose (Biozyme) preparative gels and melted at 55°C in 1.5 μl Eppendorf tubes on a thermo-block. Four microliters of liquidized PCR-product + agarose then served as template in a 2^nd^ PCR, otherwise identical to the 1^st^ but comprising only 30 cycles.

PCR-products for all unique haplotypes inferred from direct sequencing with primers Must1F/Must1R, were frozen at -20°C, immediately after PCR, subsequently cloned using the TOPO®TA Cloning®Kit and pCRTMII-TOPO® vector (Invitrogen), following the manufacture’s protocols, and then re-amplified by colony-PCR using the universal vector-based primers M13F/M13R. At least twelve successfully amplified clones per sample were then Sanger-sequenced using the nested vector-based primers T7/SP6, aligned to each-other, edited by eye for very rare singletons (explicable by PCR-errors) and reduced to the two most frequently occurring allelic VKORC1-haplotypes, as expected for diploid organisms (edition made assuming that multiple clones of the same genotype present a true sequence allele).

### Sequence evaluation and analyses

Chromatograms of Sanger sequences of mustelids were visualized using the programs Geneious (Biomatters) and/or Sequencher v.4.9, aligned to wildtype exon 3 sequences and adjacent gene regions of humans, brown rat (*Rattus norvegicus*), and house mouse (*Mus musculus*) as available from www.ensembl.org. For direct sequences, heterozygote single nucleotide polymorphisms (SNPs) were primarily detected by clear double peaks at a certain position.

### Statistical analyses

Statistical analyses were performed using R v. 3.5.1 [50]. The amount of ARs hereafter called the sum of anticoagulant residues, was calculated as the sum of coumatetralyl, bromadiolone and difenacoum residues and analyzed using generalized linear models (GLM) with gamma family and log link for strictly positive continuous data. We excluded nine samples with zero amount of AR from the statistical analysis since our focus was to detect patterns in genotypes that point towards resistance to anticoagulant rodenticides. If an animal was not exposed to ARs the information on the genotype is statistically of minor importance. First, a global model was fitted with the nonsynonymous polymorphisms and species as explanatory variables. We could not test for multiplicative effects due to the incomplete factorial design (i.e. many combinations of the nonsynonymous polymorphisms factor levels were not found). A multi-model inference approach [51] was used to fit several candidate models with different sets of explanatory variables. Species was always included as explanatory variable to account for different detection and correction methods as described above. Candidate models were compared using the Bayesian Information Criterion (BIC) and the package *MuMIn* [52]. The Akaike weight (*w*_*i*_), the second order Akaike information criterion, corrected for small sample sizes (AICc), and the Pseudo R^2^ are also reported. Variables’ importance was calculated as the sum of Akaike weight (*w*_+(j)_) across all models [51]. It is determined for each predictor variable *j* by summing the Akaike weights *w*_*i*_ across those models in the set, where the predictor variable j occurs. The models with the lowest BIC and those models within dBIC < 2, relative to the best model, were used for interpretation. Using the package *emmeans* [53], estimated marginal means, 95% confidence intervals and posthoc tests for pairwise comparisons at an alpha level of 0.05 with *p-*value adjustment by the Tukey method were performed. Model diagnostics was performed by plotting deviance residuals against fitted values and explanatory variables using the package *ggfortify* [54]. Residuals of models were not spatially autocorrelated (Moran’s I test, with *p-*values of the interpreted models > 0.81).

### Ethics statements

None of the animals analyzed in the study were killed for research purposes. They were accidentally killed by cars, domestic cats or legally culled according to the hunting regulations of the Danish Environmental Agency. The carcasses were collected by Aarhus University for research purposes (BEK no. 330, 19/03/2013 and earlier statutory orders) via the general public and private taxidermists [44,45].

## Results

### VKORC1 polymorphisms

**Fig 2:**
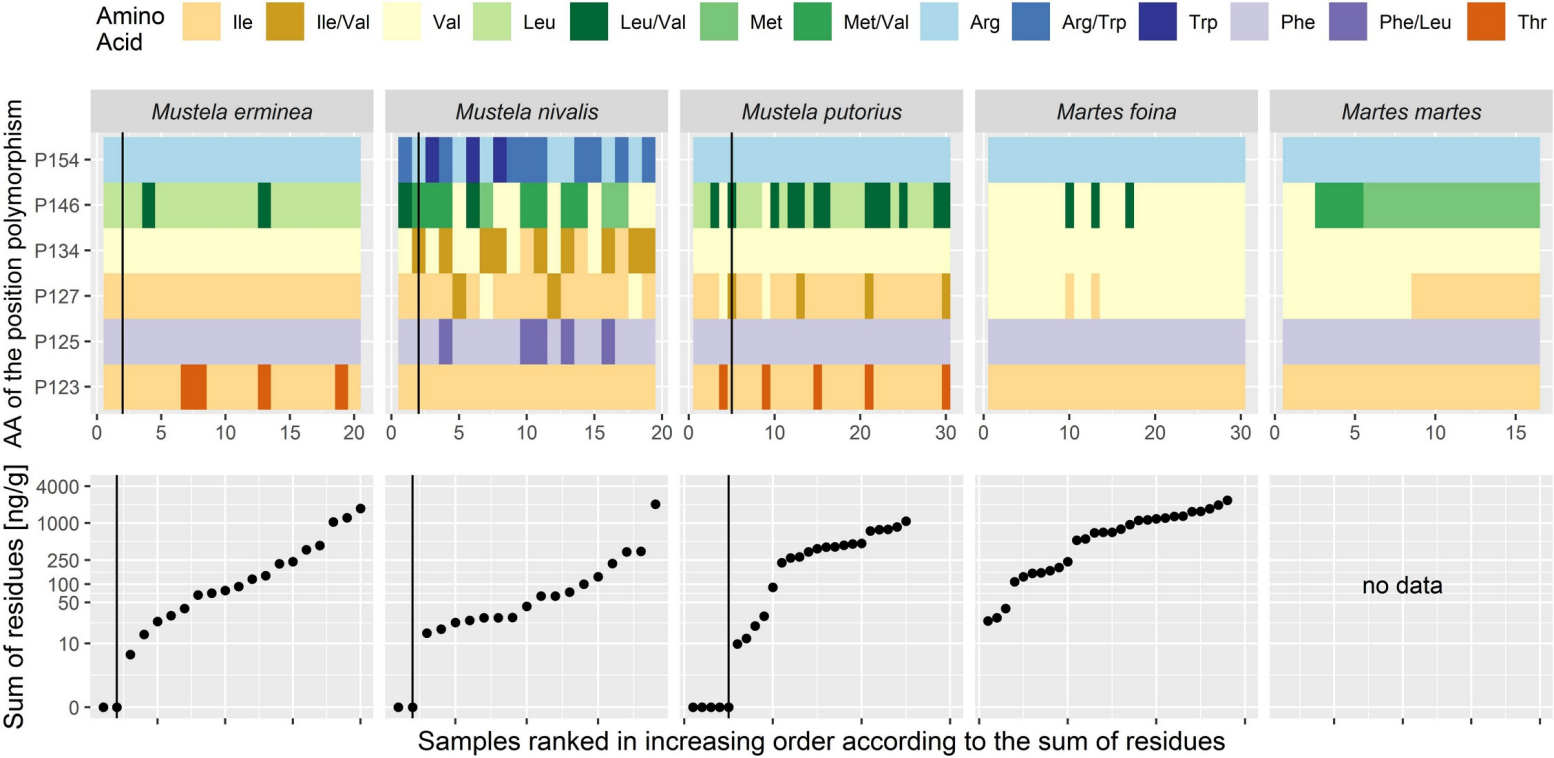
Amino acids at six selected polymorphic sites in VKORC1 (exon 3), detected in five mustelid species. Individual *Mustela erminea*, *M*. *nivalis*, *M*. *putorius* and *Martes foina* are arranged in increasing order according to the sum of anticoagulant rodenticides (AR)-residues, which are displayed below. The vertical black lines separate individuals with zero sum from those with detected residues. No AR-data were available for *Martes martes*; amino acids are labelled according to the official three letter code.

In total, we have obtained sequence information for VKORC1 from 115 tissue samples of the five mustelid species (S4 Table; S1 Data; in five mustelids, low DNA quality prevented successful amplification). Cloning was applied to a small subset of PCR products and confirmed sequences inferred from direct sequencing. Polymorphisms were detected in nine AA-positions, nonsynonymous polymorphisms in the six AA-positions: 123, 125, 127, 134, 146, 154 and synonymous polymorphisms in the same and further three positions: 137, 149 and 155 (S4 Table; S1 Data). In all five species, isoleucine and phenylalanine were most the frequent amino acids in positions 123 and 125, respectively (Table 2; Fig 2). Furthermore, at these positions we found homozygote states with two copies for tyrosine and heterozygote genotypes, where leucine co-occurred with another amino acid, respectively. In position 127, isoleucine was the most frequent amino acid in all *Mustela* but also occurred in *Martes* species. Additionally, valine was found in homo- and heterozygote genotypes. In AA-position 134, all examined mustelid species exhibited valine and in position 154 argenine, except for *Mustela nivalis*. In *Mustela nivalis*, however, we found individuals with homo- and heterozygote allele combinations involving isoleucine in position 134 as well as with homo- and heterozygote genotypes involving tryptophan. Three different amino acids (valine, leucine, methionine) occurred under homo- or heterozygote states in the AA-position 146. *Mustela nivalis* was characterized by the highest AA-variability in position 146, only homozygote leucine has not been found.

**Table 2:** Non-synonymous amino acid polymorphisms in exon 3 detected in the five mustelid species. Four polymorphisms (#17, 18, 22, 24) were found in more than one species. For synonymous polymorphisms see S5 Table.

	Number of	123		125		127			134			146					154			Polymorphism number
Species	individuals	Ile	Thr	Phe	Phe/Leu	Val	Val/Ile	Ile	Val	Val/Ile	Ile	Val	Val/Leu	Leu	Val/Met	Met	Trp	Trp/Arg	Arg	
*M*. *nivalis*	1	x		**x**		**x**				**x**		**x**							**x**	1
	1	x		**x**		**x**				**x**						**x**			**x**	2
	2	x		**x**			**x**		**x**			**x**							**x**	3
	2	x		**x**				**x**	**x**			**x**						**x**		4
	1	x		**x**				**x**	**x**				**x**				**x**			5
	1	x		**x**				**x**	**x**				**x**					**x**		6
	1	x		**x**				**x**	**x**						**x**		**x**			7
	1	x		**x**				**x**		**x**		**x**						**x**		8
	1	x		**x**				**x**		**x**		**x**					**x**			9
	1	x		**x**				**x**		**x**					**x**				**x**	10
	1	x		**x**				**x**			**x**				**x**			**x**		11
	1	x		**x**				**x**			**x**					**x**		**x**		12
	2	x			**x**			**x**		**x**					**x**			**x**		13
	1	x			**x**			**x**		**x**					**x**				**x**	14
	1	x			**x**			**x**		**x**						**x**			**x**	15
	1	x			**x**			**x**			**x**				**x**			**x**		16
sum	19	19		14	5	2	2	15	7	9	3	7	2		7	3	3	9	7	
*M*. *putorius*	15	**x**		x				**x**	x					**x**					x	17
	8	**x**		x				**x**	x				**x**						x	18
	2		**x**	x			**x**		x				**x**						x	19
	2		**x**	x		**x**			x			x							x	20
	2	**x**		x			**x**		x				**x**						x	21
	1		**x**	x				**x**	x				**x**						x	22
sum	30	25	5	30		2	4	24	30			2	13	15					30	
*M*. *erminea*	15	**x**		x				x	x					**x**					x	17
	1	**x**		x				x	x				**x**						x	18
	1		**x**	x				x	x				**x**						x	22
	3		**x**	x				x	x					**x**					x	23
sum	20	16	4	20				20	20				2	18					20	
*M*. *foina*	2	x		x				**x**	x				**x**						x	18
* *	27	x		x		**x**		** **	x			**x**							x	24
	1	x		x		**x**		** **	x				**x**						x	25
sum	30	30		30		28		2	30			27	3						30	
*M*. *martes*	2	x		x		**x**	** **	** **	x			**x**	** **	** **	** **	** **			x	24
* *	3	x		x		**x**	** **	** **	x			** **	** **	** **	**x**	** **			x	26
	3	x		x		**x**	** **	** **	x			** **	** **	** **	** **	**x**			x	27
	8	x		x		** **	** **	**x**	x			** **	** **	** **	** **	**x**			x	28
sum	16	16		16		**8**	** **	**8**	16			2			3	11			16	
	115	**106**	9	**110**	5	40	6	**69**	**103**	9	3	**38**	20	33	10	14	3	9	**103**	

Overall, the highest variability of amino acid polymorphisms was detected in *Mustela nivalis*, comprising 16 out of the 28 polymorphisms as found in all mustelid samples (Fig 2). Four polymorphisms occurred in more than one species, three other polymorphisms (Table 2: last column #17, 18, 22, 24) in two species and one (18) in the three species *Mustela putorius*, *Mustela erminea* and *Martes foina*.

### Residues of anticoagulant rodenticides

**Fig 3:**
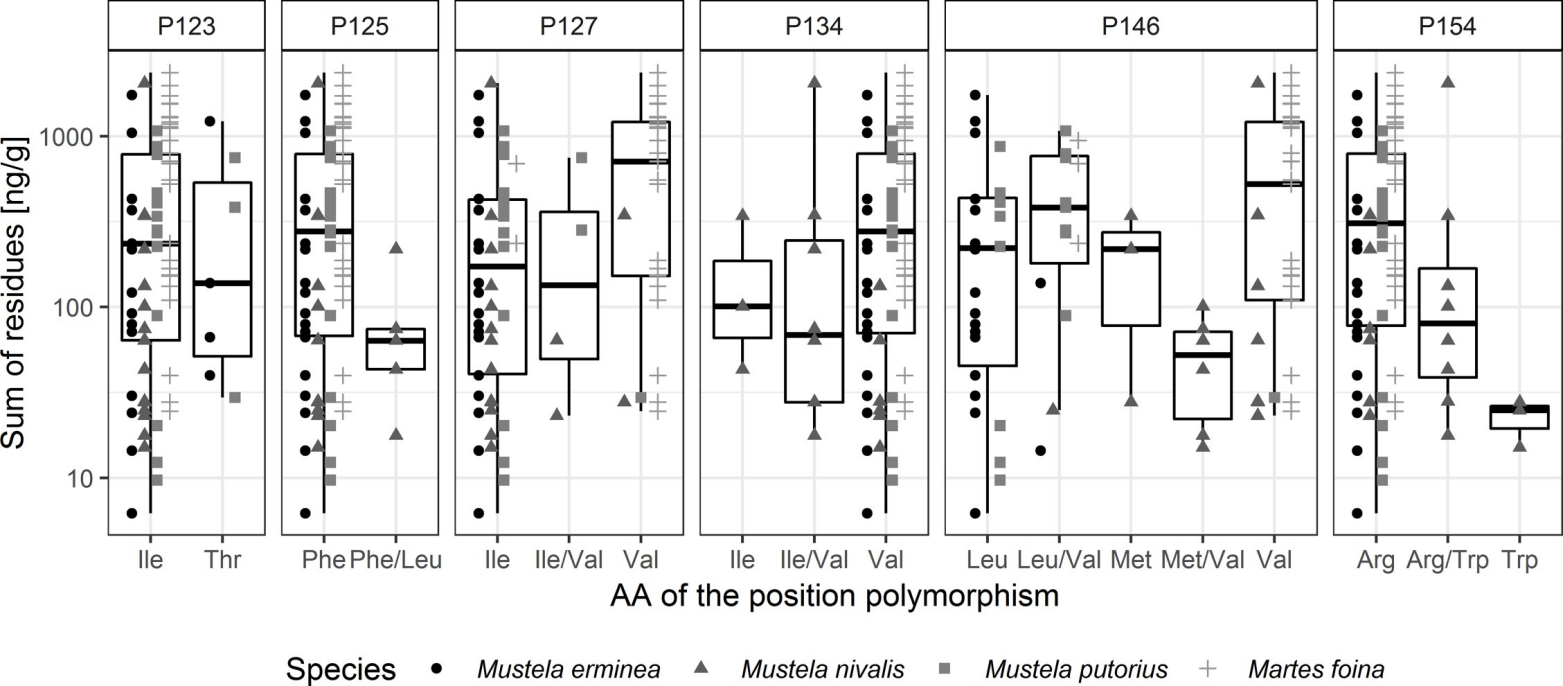
Sum of the anticoagulant rodenticide (AR-) residues of coumatetralyl, bromadiolone and difenacoum [ng/ g wet weight liver] in individuals vs. six nonsynonymous polymorphisms and four mustelid species (AA: Amino acid).

VKORC1-polymorphisms could be analysed in 115 samples (the missing five samples had to be excluded due insufficient DNA quality), for 92 samples AR residues were available, of which 83 showed detectable values (Fig 2). Their amounts varied between the four species and among the six inferred nonsynonymous polymorphisms (Fig 3). The nonsynonymous polymorphisms that best explained the sum of anticoagulant residues were (in decreasing order based on the sum of Akaike weight): P134 (w_+(P134)_ = 0.61), P125 (w_+(P125)_ = 0.55), P154 (w_+(P154)_ = 0.30), P123 (w_+(P123)_ = 0.09), P127 (w_+(P127)_ = 0.02) and P146 (w_+(P146)_ = 0.01). Comparisons yielded two models with considerable statistical support by a dBIC < 2 (Table 3). The best model indicated a higher sum of residues in Phe *vs*. Phe/Leu in P125 and a higher sum of residues in Ile/Val *vs*. Val in P134. Ile did not differ from Ile/Val and Val in P134. In the samples examined here, the lowest sum of residues was found in *Mustela nivalis*, i.e. lower than in *M*. *erminea*, *M*. *putorius* and *Martes foina* (Table 4). The second-best model indicated higher sum of residues in Arg/Trp *vs*. Trp in P154, which both did not differ from Arg. The effect of species was similar to the best model.

**Table 3:** Summary of the best fitting candidate models (dBIC < 2) and the null- and the global model to explain the sum of anticoagulant residues by non-synonymous VKORC1-mutations and species in 83 AR positive mustelids.

Models	Explanatory variables	BIC	dBIC	Akaike weight	AICc	R^2^	df
**Top 1 candidate model**	P125 + P134 + Species	1188.6	0.0	0.35	1171.2	0.31	8
**Top 2 candidate model**	P154 + Species	1190.3	1.7	0.15	1174.8	0.26	7
**Candidate model with species only**	Species	1191.1	2.5	0.10	1179.8	0.17	5
**Null model**	1	1193.1	4.5	0.02	1188.4	0.00	2
**Global model**	P123+ P125 + P127+ P134 + P146 + P154 + Species	1221.2	32.6	0.00	1189.5	0.37	17

BIC = Bayesian information criterion, dBIC = delta BIC to the best model, AICc = second order Akaike information criterion corrected for small sample size, R^2^ = pseudo-R^2^ of the model, df = degrees of freedom.

**Table 4:** Estimated marginal mean (EMM), lower (LCI) and upper confidence interval (UCI) of the sum of anticoagulant residues for each explanatory variable in the models. Results from posthoc test: different letters indicate significant differences for pairwise comparisons at alpha 0.05 with p-value adjustment of the Tukey method.

	Explanatory variables	EMM	LCI	UCI	Posthoc test
**Top 1 candidate model**					
	P125				
	Phe	1052	523	2119	a
	Phe_Leu	172	58	510	b
	P134				
	Ile	532	136	2078	ab
	Ile_Val	1334	504	3533	a
	Val	108	55	215	b
	** Species**				
	*Mustela erminea*	523	210	1299	a
	*Martes foina*	1277	538	3030	b
	*Mustela nivalis*	76	42	139	c
	*Mustela putorius*	642	262	1576	ab
**Top 2 candidate model**					
	P154				
	Arg	341	250	465	ab
	Arg_Trp	939	323	2734	a
	Trp	61	14	269	b
	** Species**				
	*Mustela erminea*	260	101	676	a
	*Martes foina*	637	260	1564	b
	*Mustela nivalis*	99	55	179	a
	*Mustela putorius*	320	125	818	ab

## Discussion

### Potential of VKORC1 polymorphisms as indicator for ongoing or substrate for future rapid evolution of resistance to anticoagulant rodenticides

Although the genetic analysis is restricted to a single key gene, to our knowledge, this study is the first to co-examine the occurrence of VKORC1 polymorphisms and anticoagulant residues in carnivores, focusing on the standing genetic variation and its potential for rapid evolution of resistance. In total, across all five mustelid species, we have found synonymous polymorphisms in nine AA-positions and nonsynonymous ones in six positions. Our results suggest that some mutations may provide resistance given the large number of nonsynonymous amino acid replacements in a functional part of this gene. Others may comprise presumably recessive heterozygotes that may nevertheless counterbalance potentially negative mutational effects [55]. Despite relationships to AR load, possibly indicating some resistance to AR, some of this variation might represent standing (and thus possibly neutral) genetic variation.

Under ARs, mustelids might be more susceptible to become traffic victims and thus, measured AR-residues cannot differentiate between recent (high) and (low) long-term exposure to ARs. Survival also depends on ingested AR dosage and compound, some of which can persist several months in survivors’ livers (t½ >200 d) [56]. Thus, AR residues in mustelid tissues may have accumulated over many months or result from recent preying on heavily poisoned rodents.

Our statistical analyses revealed that differences in anticoagulant residues in mustelids (as measured in liver tissues) may depend on the possession of specific polymorphisms. For example, polymorphisms in positions 134, 125 and 154 were associated with the sum of anticoagulant residues. Given the exploratory nature of the data and the occurrence *vs*. absence of certain nonsynonymous polymorphisms in some species (Fig 2), our statistical results must be interpreted with caution. In addition, due to the incomplete factorial design (not all possible amino acid polymorphisms co-occurred in all positions), epistatic effects could not be assessed (see also Material and Methods). As stated, we excluded few (nine) samples with zero AR from the statistical analysis as we aimed at detecting genotypes that point towards resistance. Without detectable AR-exposure, genotype information is less meaningful, as we do not know whether animals may have survived previous exposure. While such case-only studies may be susceptible to bias, a meta-analysis [57] has shown it to be negligible compared to case-control studies and case-only designs may prove of considerable value. In our study, a case-control design would require more animals with different genetic background and data on exposure to ARs as well as survival, which is not feasible in wild animals.

The structure of the protein encoded by VKORC1 in the endoplasmic reticulum of mammalian cells [17,18] contains two important amino acid motifs, encoded by exon 3, that have been biochemically well characterized: i) the CIVC-motif, with the amino acids cysteine, isoleucine, valine and cysteine, and ii) the TYA-motif, with the amino acids threonine, tyrosine and alanine. The CIVC motif (amino acid positions 132–135), plays an important role in reducing vitamin K epoxide to vitamin K [58], which is then catalyzed into its active form, vitamin K hydroquinone [59]. The TYA-motif (positions 138–140) was identified as a binding site for warfarin/coumarin [60], where mutations lead to a resistance [61]. However, even in mammals including humans, the 3D-structure of the VKORC1 is still under discussion [18]. Therefore, it remains partly speculative whether the VKORC1-polymorphisms found in exon 3 of the mustelids may indeed facilitate resistance since we have currently no mustelid-specific proof for their functional effects. However, several of the mutations clearly sit in the protein within the membrane of the endoplasmic reticulum and in close vicinity of known resistance-inducing mutations. In brown rats and house mice, both homo- and heterozygote replacements of Tyr by Cys in position 139 are responsible for resistance to the substances warfarin, coumatetraly, chlorophacinon, bromadiolone and difenacoum [30,61].

Especially in *Mustela nivalis*, the SNPs in position 134 (valine/isoleucine) resides in the CIVC-motif, and there, mutations may result in lowering its function as a catalyst. At position 146 (valine/leucin or valine/methionine) another SNP was found within the membrane in all mustelids species. A SNP detected exclusively in *Mustela nivalis* at amino acid position 154 (arginine/tryptophan) seems to be located in the C-terminus of the protein and thus in the cytoplasm, i.e. at the site of action of the anticoagulants, which may have partial functional relevance. Blood clotting tests, as described for rats [62,63] would be one way of testing the functional relevance of VKORC1 polymorphisms in the future.

### Known patterns of anticoagulant selection, susceptibility and resistance in mammals

In resistant rats, which exhibit increased vitamin K food requirements and reduced litter sizes as consequences of their resistance [64,65], there are indications for selection against such resistant individuals, when exposure to the toxin (bromadiolone) is removed. Under bromadiolone regime, however, their increased tolerance suggested that continuous anticoagulant selection would increase the proportion of highly resistant rats in the population [66]. In addition, resistance that allows survival of higher dosages of anticoagulants, can nevertheless be associated with intoxication effects. In non-target species, secondary exposure to ARs may lead to death from hemorrhaging. Despite the observed presence of VKORC1 polymorphisms that might indicate increased AR tolerance, there is an inverse relationship between AR-concentrations and general body condition in *Mustela erminea* and *Mustela nivalis* in the examined populations [41]. Persistent sublethal exposure in humans and rats include specific pathologies such as arterial calcification, severe skin irritation and both immune activation and suppression [67 and refs. therein). In conclusion, mutations in VKORC1 may be sometimes under balancing selection, favoring wild-type and resistance [55].

Secondary exposure of non-target species of rodenticides can occur by feeding on poisoned baits or along the food chain, i.e. by feeding on poisoned prey. Several studies provided experimental evidence that ARs can cause the death of mustelides by secondary exposure (*Mustela nivalis*, Warfarin: [46]; *Mustela erminea*, Bromadiolone: [47,68]) and have suggested bromadiolone to cause mustelid decline from abundances in field, however, without analyzing AR residues. Rodent control with brodifacoum in New Zealand resulted in the death of *Mustela erminea* and *M*. *putorius* (liver AR: 1000–2000 ng/g) [69]. In our study, brodifacoum levels in individuals were lower than 500 ng/g, and it is hard to know whether these present sublethal dosages. It seems likely that the exposure of the five examined predators in Denmark to these rodenticides is a result of secondary exposure through feeding on poisoned prey. For *Mustela erminea*, *Mustela nivalis* and *Mustela putorius*, this seems very probable, due to their high amount of rodent prey. The martens (*Martes martes*, *Martes foina*) though appear to have a broader food spectrum and may thus also occasionally feed on poisoned baits. Nevertheless, in Denmark, rodenticides baits should not be applied where martens and other non-target species have access; therefore, in practice, direct consumption appears negligible as a source of the detected anticoagulants [44,45].

## Conclusions

Across all five mustelid species, we found nonsynonymous (= amino-acid-change-causing) exon-3-polymorphisms in six positions and several synonymous polymorphisms in all species. Statistical analyses suggested tolerance to AR-exposure depends on species and certain amino acid polymorphisms. While several polymorphisms may present standing (and thus potentially neutral) genetic variation, some are situated in membrane parts of the VKORC1-encoded protein and might have the potential for rapid evolution of resistance in the population if the advantage of AR resistance outweighs the costs of the resistance—as seen in rats. The new molecular markers may allow quantifying indirect effects of ARs on rodent predators.

Our results, however, can only be a first indication for the role of polymorphisms, anticoagulant residues and potential tolerance, but we are far from fully understanding the potential costs of these VKORC1 polymorphisms on individual mustelids’ fitness and on the species’ conservation status. Future research is required to fully understand the genomic, toxicological and conservation consequences of secondary AR-exposure.

## Supporting information

S1 Table Nonsynonymous exon 3 specific VKORC1-polymorphisms in humans, rodents and mustelids. (XLSX)

S2 Table Sample information of the individual mustelids. (XLSX)

S3 Table Hepatic anticoagulant rodenticide concentrations (ng/g wet weight) in the analyzed mustelids (S3a corrected values; S3b uncorrected values). (XLSX)

S4 Table Individual sequences of VKORC1 (exon 3) of mustelids from this study in comparison with sequences of humans, mice, and rats. (XLSX)

S5 Table Synonymous and nonsynonymous VKORC1 polymorphisms detected in cloned sequences and synonymus VKORC1 polymorphisms detected in direct sequences of the five mustelid species. (DOCX)

S1 Data DNA sequences of VKORC1 (exon 3) of mustelids of mustelids (this paper); aligned fasta formatted file involving the IUPAC codes for nucleotides. (FA)
