# Supplementary material for: The potential of VKORC1 polymorphisms in Mustelidae for evolving anticoagulant resistance through selection along the food chain
Source: PLoS One. 2019 Aug 29;14(8):e0221706. doi: 10.1371/journal.pone.0221706 (PMC6715177; doi:10.1371/journal.pone.0221706)
Supplement: S5 Table — (DOCX) [file pone.0221706.s005.docx]

S5 Table. Synonymous and nonsynonymous VKORC1 polymorphisms detected in cloned sequences and synonymous VKORC1 polymorphisms detected in direct sequences of the five mustelid species.

| ***source*** | ***Martes martes*** | ***Martes foina*** | ***Mustela nivalis*** | ***Mustela erminea*** | ***Mustela putorius*** |
| --- | --- | --- | --- | --- | --- |
| cloned sequence |  |  | Arg135Cys |  |  |
|  |  |  | Phe150Phe |  |  |
| direct sequence |  |  |  | Ile123Ile | Ile123Ile |
|  |  |  | Phe125Phe |  |  |
|  |  |  | Ile127Ile |  | Ile127Ile |
|  |  |  | Val127Val |  | Val127Val |
|  |  |  | Ile134Ile |  |  |
|  |  |  | Val134Val |  |  |
|  | Thr137Thr | Thr137Thr | Thr137Thr | Thr137Thr | Thr137Thr |
|  | Met146Met | Val146Val | Met146Met | Leu146Leu | Leu146Leu |
|  | Val146Val |  | Val146Val |  | Val146Val |
|  |  |  | Ser149Ser |  |  |
|  |  |  | Arg154Arg |  |  |
|  |  |  | Trp154Trp |  |  |
|  |  |  |  |  | Glu155Glu |
